# Connectivity-Based Predictions of Hand Motor Outcome for Patients at the Subacute Stage After Stroke

**DOI:** 10.3389/fnhum.2016.00101

**Published:** 2016-03-09

**Authors:** Julia Lindow, Martin Domin, Matthias Grothe, Ulrike Horn, Simon B. Eickhoff, Martin Lotze

**Affiliations:** ^1^Functional Imaging Unit, Center for Diagnostic Radiology, University of GreifswaldGreifswald, Germany; ^2^Institute of Neurology, University of GreifswaldGreifswald, Germany; ^3^Institute of Clinical Neuroscience and Medical Psychology, Heinrich Heine University DüsseldorfDüsseldorf, Germany; ^4^Institute of Neuroscience and Medicine (INM-1), Research Centre JülichJülich, Germany

**Keywords:** diffusion weighted imaging, motor outcome, prediction, recovery, resting state fMRI, stroke

## Abstract

**Background**: Connectivity-based predictions of hand motor outcome have been proposed to be useful in stroke patients. We intended to assess the prognostic value of different imaging methods on short-term (3 months) and long-term (6 months) motor outcome after stroke.

**Methods**: We measured resting state functional connectivity (rsFC), diffusion weighted imaging (DWI) and grip strength in 19 stroke patients within the first days (5–9 days) after stroke. Outcome measurements for short-term (3 months) and long-term (6 months) motor function was assessed by the Motricity Index (MI) of the upper limb and the box and block test (BB). Patients were predominantly mildly affected since signed consent was necessary at inclusion. We performed a multiple stepwise regression analysis to compare the predictive value of rsFC, DWI and clinical measurements.

**Results**: Patients showed relevant improvement in both motor outcome tests. As expected grip strength at inclusion was a predictor for short- and long-term motor outcome as assessed by MI. Diffusion-based tract volume (DTV) of the tracts between ipsilesional primary motor cortex and contralesional anterior cerebellar hemisphere showed a strong trend (*p* = 0.05) for a predictive power for long-term motor outcome as measured by MI. DTV of the interhemispheric tracts between both primary motor cortices was predictive for both short- and long-term motor outcome in BB. rsFC was not associated with motor outcome.

**Conclusions**: Grip strength is a good predictor of hand motor outcome concerning strength-related measurements (MI) for mildly affected subacute patients. Therefore additional connectivity measurements seem to be redundant in this group. Using more complex movement recruiting bilateral motor areas as an outcome parameter, DTV and in particular interhemispheric pathways might enhance predictive value of hand motor outcome.

## Introduction

Stroke is the leading cause of adult disability worldwide, leaving a majority of patients with lingering upper limb impairment (World Health Organization, 2012). Knowing more about motor outcome would be advantageous to achieve the best results in rehabilitation. For long-term motor outcome prediction, parameters are needed that can be assessed within the first days, when patients are in the acute care units.

Functional magnetic resonance imaging (fMRI) raised high expectations because functional representation of movements can be assessed longitudinally *in vivo*. However, activation fMRI protocols are demanding and patients’ compliance is difficult to control for. In contrast, resting state fMRI (rs-fMRI) requires little compliance and can therefore be conducted comparable to structural MRI in the acute (0–24 h after stroke onset) to subacute (24 h to 6 weeks after stroke) phase after stroke (Di Pino et al., 2014). Especially rs-fMRI functional connectivity (FC) between cortical motor areas has been described to be associated with motor impairment (Carter et al., 2010). Astonishingly, only a low number of studies examined the prognostic value of resting-state functional connectivity (rsFC) for motor outcome in acute stroke patients.

To date the best predictor of later hand motor outcome is the initially measured hand motor impairment. Especially the Fugl-Meyer test (Sanford et al., 1993) has been described as a valuable predictor of hand motor outcome for 2, 6 and 12 months after stroke for mildly to moderately impaired patients (Feys et al., 2000). Likewise the active motion range is known to be a good predictor of short-term (3 months; Beebe and Lang, 2009) and long-term (6 months; Smania et al., 2007) hand motor outcome.

In more severely affected stroke patients, the intactness of the corticospinal tract (CST), as tested with diffusion weighted imaging (DWI) is useful for the prediction of hand motor outcome (Lindenberg et al., 2012; Stinear et al., 2012; Groisser et al., 2014; Byblow et al., 2015). TMS-measures such as the asymmetry index of hand muscle motor evoked potentials is a clinically well suited predictive method for describing motor outcome in patients after stroke (e.g., Stinear et al., 2012; Byblow et al., 2015). At the subacute phase, a positive association between fractional anisotropy (FA) measured at the height of the posterior limb of the internal capsule of the ipsilesional side and hand motor performance has been shown (Jang et al., 2005; Konishi et al., 2005; Nelles et al., 2008; Byblow et al., 2015). Most authors used FA as well as axial and radial diffusivity for quantification of intactness of the CST. When comparing the predictive value of these measurements, differences in axial diffusivity of the pyramidal tract from the ipsilesional to the contralesional hemisphere at the acute phase had the highest association with 3 and 6 months motor outcome of grip strength and nine hole peg test (NHPT) in a sample of 10 initially strongly impaired stroke patients (Groisser et al., 2014). Probabilistic tractography methods using the DWI data are capable of reconstructing diffusion pathways over long distances even when fibers are crossing (Lindenberg et al., 2010). We thought that the possibility to find alterations among long anatomical pathways might be advantageous for predicting motor outcome even in less severely impaired patients.

Carter et al. (2012a,b) assessed connectivity approaches as a promising method for understanding the impact of cerebral lesions on motor function and its restitution. Consequently, they combined DWI of the CST with FC measurements as assessed by rs-fMRI. For rs-fMRI positive associations with motor performance at the chronic phase after stroke (>6 weeks after stroke; Di Pino et al., 2014) have been described between homotopic motor areas of the affected and the unaffected side indicating that more “balanced” activity between hemispheres is associated with better upper-limb control (Urbin et al., 2014). For rs-fMRI two studies describe a positive association between homolog motor areas between both hemispheres and motor performance (Carter et al., 2010, 2012a). However, both did not measure future motor outcome using resting state connectivity but performed only correlative measurements assessed at about the same time. Overall, stroke patients with motor impairment show decreased interhemispheric M1-connectiviy and increased resting-state connectivity between ipsilesional M1 and secondary motor areas particularly in the ipsilesional hemisphere (Rehme et al., 2015). Over a period of 3 months the reduced interhemispheric M1 rsFC normalizes (Golestani et al., 2013). To date only one study has applied longitudinal motor outcome measurements to investigate the value of rsFC for predicting motor outcome. Park et al. (2011) investigated rs-fMRI in 12 subacute stroke patients and found a positive association between 6 months motor outcome measured with Fugl-Meyer-Score and rsFC of the ipsilesional M1 with the contralesional thalamus, supplementary motor area (SMA), and medial frontal gyrus.

The present study examined the prognostic value of motor (grip strength, NHPT), and clinical (NIH stroke scale; NIHSS) scores, DWI of long tracts and rs-FC for patients at the subacute stage with predominantly only mild unilateral brain damage. We used two different motor outcome scores: the motricity index (MI) for upper limb and the box and block test (BB) to examine separate aspects of upper limb function namely strength (MI), and hand grip transfer (BB). We hypothesized that intactness of long tracts, would be a predictor for both scores. However, hand strength is represented unilaterally, whereas grip transfer recruits bilateral resources from both hemispheres (Lotze et al., 2012). More bilateral activation might involve increased information transfer (inhibitory or excitatory) via the corpus callosum between both primary motor cortices (M1). Therefore we hypothesized that integrity of interhemispheric fibers would be better predictors for BB, whereas CST integrity might be a better predictor for MI. In addition, we expected lower rsFC between ipsilesional primary motor cortex and contralesional secondary motor areas (SMA, dorsal premotor cortex, dPMC) to be associated with better motor outcome (MI and BB; Wang et al., 2010).

## Materials and Methods

### Participants

Twenty-four stroke patients [aged 67.7 ± 8.7 years (mean ± standard deviation), 11 female, 19 right handed] were recruited through the stroke unit at the Department of Neurology of the University Medicine Greifswald.

**Figure 1:**
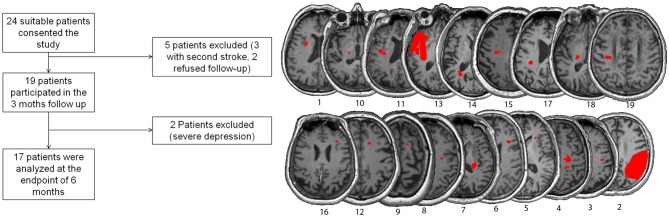
**Left:** Inclusion/exclusion flowchart. **Right:** Lesion maps of each participant with lesions indicated in red.

Inclusion criteria were: (1) first ischemic stroke; (2) unilateral upper limb impairment at day 2 after stroke; (3) no contraindications for MRI; (4) older than 18 years; and (5) being able to consent for study participation at day 5 after stroke. Exclusion criteria were: (1) global aphasia; (2) cognitive impairment; and (3) other neurological diseases. Overall, 19 patients were included in the 3 months survey and 17 in the 6 months survey (see Table 1 and Figure 1).

**Table 1:** **Patient characteristics**.

Patient	Age at assessment (years)	Gender	Affected hemisphere	Lesion size (cc^3^)	Lesion location	NIHSS subacute	Grip strength subacute affected side (bar)	NHPT subacute affected side (pegs/sec)
1	72	F	L	0.34	sc, internal capsule, putamen	1	*	0.33
2	72	M	R	60.63	c/sc arteria media region	4	0.36	0
3	70	M	R	0.27	sc, internal capsule	1	0.56	0.14
4	61	M	R	1.23	sc, frontal	1	0.76	0.34
5	53	M	R	0.08	sc, putamen	2	0.76	0.37
6	70	F	R	0.32	c, M1 and PMC	1	0.64	0.36
7	76	M	R	1.31	sc, internal capsule	2	0.51	0.28
8	60	F	R	0.06	sc, frontal	0	0.79	0.42
9	70	F	R	0.23	sc, frontal	1	0.53	0.44
10	78	F	L	0.20	sc, internal capsule	0	0.57	0.39
11	72	F	L	0.21	sc, pyramidal tract	2	0.50	0.35
12	62	M	R	0.16	sc, frontal	3	0.54	0.39
13	60	M	L	30.30	c, temporal pole	1	0.89	0.39
14	51	M	L	0.53	sc, parietal	2	0.55	0.41
15	76	M	L	0.38	sc, pyramidal tract	2	0.96	0.45
16	64	F	R	0.01	sc, frontal	2	0.44	0.49
17	74	M	L	1.18	sc, pyramidal tract	1	0.73	0.38
18	64	F	L	0.56	sc, int. capsule	3	0.58	0.26
19	49	M	L	0.47	sc, int. capsule, putamen	2	0.42	0.19
Mean	69.67	8 F/11 M	9 L/10 R	5.18		1.63	0.62	0.34

Abbreviations: F, female; M, male; L, left; R, right; NIHSS subacute measured during the first 5 days; c, cortical; sc, subcortical; int. capsule: internal capsule. *Measurement not possible because of initial injury.

All subjects gave written informed consent to attend the study, which was approved by the National Ethics Committee of the German Society for Psychology (DPG).

### Clinical Measures

Stroke severity was determined by the National Institutes of Health Stroke Scale (NIHSS) that ranges from 0 (no deficits) to 42 (severe stroke) at the time of hospitalization (NIHSS initial), at inclusion (NIHSS subacute), at 3 months (NIHSS 3 months) and 6 months (NIHSS 6 months) post-stroke (Brott et al., 1989).

The “Montreal Cognitive Assessment Test” (MoCA) was used to screen for cognitive abilities in various domains: visuo-spatial and short-term memory, attention, language, abstraction, executive function, and orientation (Dong et al., 2010). The Beck-Depression-Inventory (BDI) was used to screen for depression (Beck et al., 1961). These global scores revealed that patients on average were moderately depressed (7.57 ± 9.41) post stroke and showed mild cognitive impairment (MoCA index after 3 months: 22.84 ± 3.30).

To investigate the predictive value of motor scores we tested two measurements of hand motor performance initially, 3 and 6 months post-stroke: hand grip strength (Martin Vigorimeter, Gebrüder Martin, Tuttlingen, Germany) and finger dexterity (nine hole peg test, NHPT). For grip strength the average value of five trials was taken as the value for grip strength for each hand (Desrosiers et al., 1995). For finger dexterity, we used the NHPT, measuring the time to put all pins into the nine holes and remove them back into the container. The NHPT score was determined by averaging over two trials (Mathiowetz et al., 1985a). Results were expressed in pegs per second (higher value means better performance).

As motor outcome variables we used the MI motricity index of the affected upper limb and the BB (Mathiowetz et al., 1985b). The MI is especially known for being a valid instrument to assess the strength of the paretic upper extremity after stroke by measuring the degree of the paresis including hand-grasp, elbow flexion and shoulder abduction (Collin and Wade, 1990). It has been applied in several studies on motor outcome in stroke patients (Kwakkel et al., 2006). The score ranges from minimum score of 0 (plegic) to the maximum of 100 (recovered). MI was acquired at baseline (subacute stage), 3 and 6 months after stroke. The BB is a valid measurement to determine unilateral gross manual dexterity (Platz et al., 2005). Complex movements of several joints are required to move as many blocks as possible from one compartment to another within 60 s. The BB was acquired at 3 and 6 months after stroke. The average number of cube transferred per minute within the age range of patients in this study is 69.

### Duration of Neurorehabilitation

We assessed the following information about the Neurorehabilitation: all patients got physical and/or occupational therapy during their hospital stay, whose average length was 9.3 ± 3.6 days. Afterwards 11 patients (64.7%) were sent to an acute neurorehabilitation clinic and the average days of therapy were 27.12 ± 11.6 days. Additionally five patients (29.4%) had an ambulant physical and/or occupational therapy when they were back at home again with an average of 22.4 ± 24.3 h. Overall, the total number of physical and/or occupational therapy days that the patient received between the hospital stay and the 6 months follow-up amounted to 27.7 ± 11.4 days.

### Magnetic Resonance Imaging (MRI)

MRI was conducted with a 3 Tesla MRI-Scanner (Verio, Siemens, Erlangen, Germany) using a 32-channel head coil from day 5 after stroke (average: 7.5 days) to avoid artifact from tissue edema affecting water diffusion. After day 5 the effect of edema was assumed to be negligible (Sotak, 2002).

#### Resting State fMRI

We used gradient echo planar imaging (EPI) of 150 whole head volumes with 36 transverse slices each, with an in plane spatial resolution of 3 × 3 mm^2^, slice thickness of 3 and 1 mm gap. The temporal resolution (TR) of each volume was 2 s. The whole rs-fMRI lasted for a period of 6 min. Participants were instructed to close their eyes, let their mind wander and avoid to mentally fixate on a certain issue.

#### T1-Weighted Cranial Imaging

An anatomical 3D T1-weighted MPRage dataset was acquired with a voxel size of 1 mm isotropic, acquiring 176 sagittal slices, with a TE of 2.5 ms and a TR of 1900 ms. The whole measurement duration was about 7 min.

#### Diffusion Weighted Imaging

We applied a Siemens MDDW (Multi Directional Diffusion Weighting) sequence with the following parameter setup: voxel size: 1.8 × 1.8 × 2.3 mm^3^, 55 slices, 1 acquisition and 64 directions. One b0-volume was measured and *b* = 1000 s/mm^2^ was used for the diffusion-weighted images. TR was 10,500 ms, TE: 107 ms and the total scan time was 12 min.

### Data Evaluation

#### DWI Processing

Gray matter parts of all cortical ROIs (MNI-space) were removed by using a white matter mask (MNI-space, provided by FSL) in order to prevent tractography to and from non-white matter structures. DWI data were coregistered to the anatomical 3D T1-weighted dataset, which in turn was spatially normalized to the MNI space using FSL FNIRT. The inverse of the resulting transformation was used to spatially denormalize the masked ROIs into subject space. DWI data were processed utilizing BEDPOSTX and PROBTRACKX of the FSL software package. First, the diffusion data were corrected for eddy current and head movement artifacts. Then, BEDPOSTX was executed to build up distributions on diffusion parameters and modeling crossing fibers at each voxel of the brain. PROBTRACKX was used to calculate a structural connectivity distribution between selected ROIs (Behrens et al., 2007). The resulting probabilistic streamlines were then normalized by region-of-interest sizes (volume of all contained voxels; Rilling et al., 2008) and thresholded with 10% of the highest connectivity value of a certain probabilistic streamline. The normalized and thresholded probabilistic streamline was evaluated with regard to its voxel volume. This value is now referred to as diffusion-based tract volume (DTV).

#### Resting State Preprocessing

Data were preprocessed using SPM8 (Wellcome Department of Cognitive Neuroscience, London, UK). Images were first corrected for head movement by affine registration using a two-pass procedure by which images were initially realigned to the first image and subsequently to the mean of the realigned images. Each participant’s mean image was then spatially normalized to the Montreal Neurological Institute (MNI) single-subject template brain using the “unified segmentation” approach and the ensuing deformation was applied to the individual EPI volumes (Ashburner and Friston, 2005). Hereby, volumes were resampled at 1.5 × 1.5 × 1.5 mm^3^ voxel size. Images were then smoothed by a 5 mm full-width at half-maximum Gaussian kernel to increase the signal-to-noise ratio and compensate for remaining differences in individual anatomy. Confound removal was performed as described before Lotze et al. (2014). After confound removal, data were band-pass filtered preserving frequencies between 0.01 and 0.08 Hz, as meaningful resting-state correlations will predominantly be found in these frequencies given that the BOLD response acts as a low-pass fimoot (Greicius et al., 2003).

#### Regions of Interest (ROIs)

We included the following ROIs: the primary motor cortex (M1) both ipsilesional (il) and contralesional (cl) as seeds for both rsFC and DTV (Carter et al., 2010, 2012a; Lindenberg et al., 2010; Lu et al., 2011; Urbin et al., 2014). In addition, we tested the long pathways from M1^il^ to the anterior cerebellar hemisphere^cl^ (cerebellar Larsell lobules H IV-VI; Cb^cl^) for both the rsFC and the DTV-analysis (for rsFC: Wang et al., 2010; Lu et al., 2011; for DWI: Groisser et al., 2014). The dPMC and SMA proper were only used as seeds for rsFC (Carter et al., 2010, 2012a; Rehme et al., 2011). In contrast, pathways descending from M1 to the height of the pons have been only included for the DTV-analysis (see also Lindenberg et al., 2010).

For our study dPMC and SMA proper were selected from the HMAT-Atlas (Human Motor Area Template, Laboratory for Rehabilitation Neuroscience, University of Florida). The seed for the primary hand motor area was generated by drawing a point into the hand knob area of both the left and right side of the brain (Yousry et al., 1997). The point represented the center for a spherical region of 7 mm radius, which was created by a dilation process. The JHU ICBM-DTI-81 White-Matter Labels (Laboratory of Brain Anatomical MRI, Johns Hopkins University) were used to extract ROIs for the descending pyramidal tract in the ipsilesional pons.

We applied probabilistic tractography and did not use conventional diffusion tractography (DTI) quantification (FA or diffusivity) since probabilistic tractography is better suited for following white matter connections over long distances (M1^il^ with the pons^il^, M1^il^ with the anterior cerebellar hemisphere^cl^) or between the primary motor cortices (M1^il^–M1^cl^; Bucci et al., 2013).

For each participant, the rsFC time-series data of each seed region were extracted and correlated with each other, and the resulting Pearson correlation coefficients were transformed into Fisher’s *Z* scores. These were then used for further statistical testing.

### Statistical Testing

**Figure 2:**
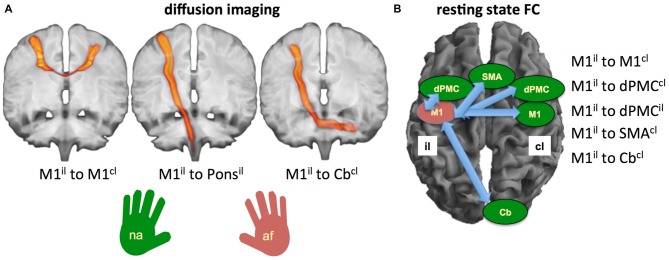
**Visualization of tracts and regions of interest (ROIs) used for diffusion-based tract volume (DTV) and rsFC analysis. (A)** Pathways included in the probabilistic DWI-tracking demonstrated with probability tracking of all participants: left: M1^il^ to M1^cl^; middle: M1^il^ to Pons^il^; right: M1^il^ to Cb^cl^. **(B)** Selected ROIs to calculate rsFC. Abbreviations: M1, primary motor cortex; dPMC, dorsal premotor cortex; SMA, supplementary motor area; Cb, cerebellum; il, ipsilesional; cl, contralesional; af, affected hand; na, non-affected hand.

We used Wilcoxon tests to determine differences between motor scores (MI and BB) between time points. The predictive value for the outcome of the MI and BB of the affected hand at 3 and 6 months was calculated in six stepwise multiple linear regression analyses of parameters assessed in the subacute phase. These comprised clinical scores (grip strength, NIHSS and NHP-test), probabilistic tracking (M1^il^–M1^cl^, M1^il^–pons^il^, M1^il^–Cb^cl^) and rsFC (Mi^il^–M1^cl^; M1^il^–dPMC^cl^, M1^il^–dPMC^il^; M1^il^–SMA^cl^, M1^il^–Cb^cl^) for the two time points each (3 and 6 months; see Figure 2).

The goodness of fit is expressed in terms of *R^2^* and significance in an *F*-test. The resulting standardized regression coefficients are given. Table 2 provides an overview on the models tested and the results.

**Table 2:** **Prediction of motor outcome parameters with stepwise multiple linear regression**.

Predictive values	Outcome parameter	
	**MI 3 months**	**MI 6 months**
Clinical measures	Grip strength	Grip strength
	(*R^2^* = 0.54)	(*R^2^* = 0.44)
Resting state	No predictive power	No predictive power
DWI	No predictive power	MI^il^–ant cerebell hem^cl^
		(*R^2^* = 0.23)
	**BB 3 months**	**BB 6 months**
Clinical measures	No predictive power	No predictive power
Resting state	No predictive power	No predictive power
DWI	M1^il^–M1^cl^	M1^il^–M1^cl^
	(*R^2^* = 0.25)	(*R^2^* = 0.28)

*Abbreviations: MI, motricity index; BB, block and box test; DWI, diffusion weighted imaging; M1, primary motor cortex; il, ipsilesional; cl, contralesional*.

## Results

**Figure 3:**
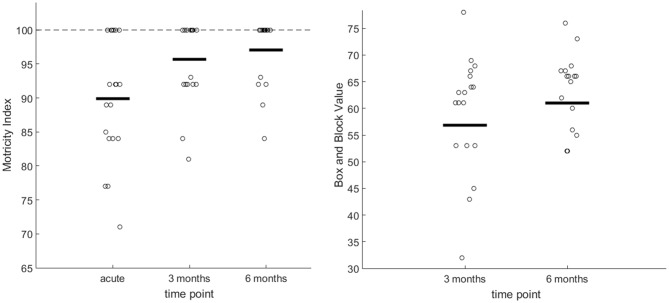
**Left graph:** Plots of all motricity index (MI) scores for the affected hand during acute, 3 and 6 months. The bold line indicates the median. The staggered line indicates the maxima. **Right graph:** Plots of the box and block (BB) test for the affected hand, 3 and 6 months after stroke. The bold line indicates the median.

**Figure 4:**
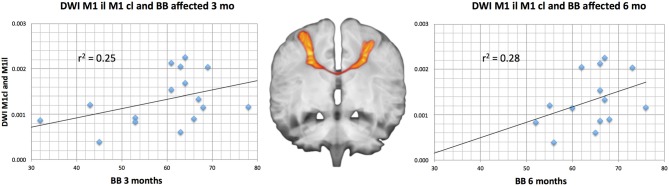
**Linear regression of significant predictive measures with the box and block test (BB) after 3 and 6 months for the M1^il^–M1^cl^ DTV.** The tract course is demonstrated on a diffusion weighted image overlaid on a T1-weighted image both measured from a healthy control.

The NIHSS score at admission was 5.32 ± 5.25 and 1.63 ± 1.01 at time of MRI measurement (average: 7.5 days). MI showed relevant changes during the short term [Wilcoxon test between baseline and 3 months: *U*_(18)_ = 2.51; *p* = 0.012] and long term period [between 3 and 6 months: *U*_(16)_ = 1.73; *p* = 0.040; see plots in Figure 3, left]. BB of the affected hand at 3 months was on average 56.84 ± 14.53 and at 6 months 61.00 ± 13.26 (see plots in Figure 4, right) with significant differences between 3 and 6 months measures (*U*_(16)_ = 2.28; *p* = 0.023).

Grip strength predicted MI after 3 months (*R^2^* = 0.54; *p* = 0.001) and 6 months (*R^2^* = 0.44; *p* = 0.008; Figure 4). NHPT and NIHSS were not suited to predict motor outcome and were discarded by the stepwise regression. In contrast, no motor outcome measurement (NIHSS, NHPT, MI), was associated significantly with BB.

DTV showed a very weak predictive value for both short-term and long-term motor outcome. For the 6-month MI outcome we found a strong trend for a predictive value for diffusion probability between M1^il^ and Cb^cl^ (*R^2^* = 0.23; *p* = 0.05). For the BB, we found both a short-term (3 months: *R^2^* = 0.25; *p* = 0.03) and a long- term (6 month: *R^2^* = 0.28; *p* = 0.03) predictive value for the M1^il^–M1^cl^ DTV tract probability (Figure 4). Table 2 provides an overview on the comparisons and their strength of association. All other DTV-values were rated as irrelevant by multiple regression analyses.

None of the rsFC values between the seeds showed any association with later motor outcome for MI or BB and were discarded by the stepwise regression.

## Discussion

In this study, we tested the predictive value of motor scores (grip strength, NHPT), the NIHSS, rsFC and DTV for short- and long-term motor outcome in subacute stroke patients with predominantly mild cerebral damage. Initial grip strength was predictive of MI. The intactness of interhemispheric tracts was predictive of BB outcome. In contrast, cerebro-spinal pathways from ipsilesional M1 to contralesional anterior cerebellar hemisphere were most important for MI outcome prediction rsFC showed no predictive value.

### The Predictive Value of Motor Assessments

Our study confirms earlier reports that strength measurements during the subacute stage have a predictive value for MI in initially mildly affected patients (Smania et al., 2007; Beebe and Lang, 2009). However, a high ceiling effect of MI already after 3 months due to the mild initial impairment in our patients restricted the usefulness of MI as an outcome variable. When predicting BB as an outcome measurement, there were no associations for strength, pinch grip or NIHSS. The above finding illustrates the importance of outcome measures for different aspects of motor performance. Aspects explained by one variable might not necessarily explain others.

### The Predictive Value of DTV

In our study, both the DTV for the long CST between ipsilesional M1 and the contralesional anterior cerebellar hemisphere and the M1 connecting interhemispheric tract showed relevant predictive value for motor outcome. It is not surprising that corticospinal integrity of the pyramidal tract form ipsilesional M1 is relevant for motor outcome. For severely affected patients, it has been demonstrated that intactness of the CST (van Dokkum et al., 2014) is a strong predictor for resolution of impairment (Byblow et al., 2015; Burke Quinlan et al., 2015) and can be used within an algorithm (Stinear et al., 2012) to inform about the potential of upper limb functional recovery. However, probabilistic tracking only for the most distant seeds (M1 to cerebellar hemisphere) showed any predictive value in our subacute stroke patients with predominantly mildly damage. This is certainly caused by the small amount of lesions observed in our patients leaving them with mild motor impairment. The intactness of the CST can be assessed with axonal or radial diffusivity (van Dokkum et al., 2014) as well as with FA (Schulz et al., 2012) within the posterior limb of the internal capsule. FA is a rather global measure since tracts from the dPMC, the SMA and M1 and S1 and parietal cortex are passing through this structure and especially those descending from M1 and dPMC correlate positively with grip strength in the chronic stage (Schulz et al., 2012). Probabilistic tractography is capable of testing long distance white matter tracts and is well suited for patients without circumscribed lesions to the pyramidal tract.

White matter integrity of non-crossing fibers between M1–M1 can predict training-induced performance gains in chronic patients (Lindenberg et al., 2012). Our findings are in accordance with this earlier result. We found a predictive value of M1^il^–M1^cl^ diffusivity for short- and long-term outcome of the BB. Especially for tasks recruiting bilateral resources such as during grasping and the transfer of objects, these interhemispheric tracts are of importance since they enable a bihemispheric coordination of sensorimotor (SM1) activation (Lotze et al., 2012).

### The Predictive Value of rsFC

In our study, rsFC had no predictive value for early or late outcomes spanning the subacute stage. For patients with motor impairment after stroke a decreased rsFC between M1^il^ and M1^cl^ in comparison to healthy age-matched controls is the most consistent finding reported in resting state studies on stroke patients (Carter et al., 2010, 2012a; Wang et al., 2010; Park et al., 2011; Golestani et al., 2013; Rehme et al., 2015). Previous studies measuring rsFC at the subacute stage found significant associations with motor performance at time of fMRI (Carter et al., 2010, 2012a). This however has no predictive value as demonstrated in our study using longitudinal performance measurements.

At least four longitudinal rs-fMRI studies have been conducted with stroke patients (Wang et al., 2010; Lu et al., 2011; Park et al., 2011; Golestani et al., 2013). Golestani et al. (2013) investigated 31 stroke patients with motor impairment within the first 24 h, after 7 and after 90 days and found that over time the reduced interhemispheric SM1 rsFC normalized. Their work is an excellent example of how variable rsFC is after stroke. This may be the reason why long-term motor outcome prediction is problematic using this measurement. In addition, a very recent study demonstrated that highest intra-participant variability was observed over extensive repetitive measurements in a healthy participant in the somato-motor regions (Laumann et al., 2015). This low intra-subject reliability may indicate a poor prognostic value.

### Limitations

Sample size is a limitation of the present study. Combining data from separate centers would be necessary to overcome this problem. It might further be advantageous to differentiate cortical and subcortical damage in a larger sample. Another limitation was the selection of patients who were mainly mildly to moderately impaired. Inclusion of more patients with severe initial impairment may produce different results but was excluded by our ethical commitment. Another limitation was that we could not perform BB at the subacute stage for reason of hygiene in an intensive care unit. In addition, it can not be excluded that edema at the early phase after stroke does affect imaging results.

## Conclusion

rsFC is strongly variable after stroke what makes any motor outcome prediction for this parameter difficult. It is quite astonishing that a predictive value of resting state connectivity has been postulated without performing longitudinal measurements. It might also be useful to explore the usefulness of including probabilistic tractography in combined outcome algorithms (Stinear et al., 2012, 2013, 2014). However, any potential improvements in prognosis would have to be weighed up against any added complexity of more advanced techniques. A simple approach may be necessary for optimal application in clinical practice.

## Author Contributions

JL performed measurements and investigations on the patients and contributed in writing the manuscript. MD evaluated and interpreted the DWI-data. MG helped with patient recruitment and with interpretation of data. UH helped with statistical evaluation. SBE provided tools for performing the resting state analysis and commented the analysis and the manuscript. ML planned the study, performed the statistics and wrote the manuscript.

## Funding

The study was funded by a grant from the German Research Community (DFG Lo795/7-1).

## Conflict of Interest Statement

The authors declare that the research was conducted in the absence of any commercial or financial relationships that could be construed as a potential conflict of interest.

## Abbreviations

BB: Box and Block test
BDI: Beck-Depression-Inventory
cl: contralesional
CST: corticospinal tract
Cb: anterior cerebellar hemisphere
dPMC: dorsal premotor cortex
DTV: diffusion based tract volume
DWI: diffusion weighted imaging
FA: fractional anisotropy
il: ipsilesional
MI: Motricity Index
M1: primary motor cortex
MoCA: Montreal Cognitive Assessment test
NHPT: nine hole peg test
NIHSS: NIH-Stroke-Scale
rsFC: resting state functional connectivity
SMA: supplementary motor area.
